# Syndemic approach to address the burden of antimicrobial resistance in Southeast Asia

**DOI:** 10.1016/j.lansea.2026.100747

**Published:** 2026-03-11

**Authors:** Saju Bhuiya, Fahmida Chowdhury

**Affiliations:** Infectious Diseases Division, International Centre for Diarrhoeal Disease Research, Bangladesh (icddr,b), Dhaka, 1212, Bangladesh

Antimicrobial resistance (AMR) is increasingly recognised as an ongoing pandemic, which occurs at the intersection of biology, inequity, health education, cultural norms, and the social practice of antimicrobial use. Recently published *WHO Global Antimicrobial Resistance Surveillance Report 2025* based on the Global Antimicrobial Resistance and Use Surveillance System (GLASS) offers a comprehensive picture of the AMR burden, reporting evidence of over 23 million bacteriologically confirmed infections from 104 countries.^1^ However, as GLASS relies on laboratory-confirmed surveillance data often from tertiary care facilities these figures likely underestimate the true population-level burden and may not be fully representative across settings. The participating countries in WHO GLASS have increased fourfold since 2016, in southeast Asia (SEA) region 90.9% of the countries participated in the GLASS platform reflecting substantial progress in the establishment of coordinated AMR surveillance systems, although persistent gaps hinder the full utility of these metrics for policy and practice.^1^

This report demonstrates an increasing trend of AMR in the SEA region with 30% of the bacterial infections reporting resistance against first-line antibiotics.^1^ The region has emerged as an epicentre of a possible “global syndemic dynamic”, where AMR is not only a biological or medical phenomenon, but an echo of entrenched inequities in terms of limited diagnostic capacity and sub-optimal health system.^2^ The burden of AMR is strongly influenced by socioeconomic condition and health system infrastructure, with higher AMR levels being observed in countries with weaker health system. There is also a strong inverse correlation between universal health coverage, income levels, and median AMR rates in bloodstream infections (r = −0.77, *p* < 0.001), further reinforcing the syndemic nature of AMR in the region.^1^

Although the report offers strong descriptive epidemiology with a focus on surveillance and stewardship, it underplays the socio-economic determinants and offers comparatively limited critique of the potential health-system failures that are contributing towards AMR. Surveillance for AMR in SEA region is confined to donor-dependent tertiary hospitals, disconnected from peripheral primary care settings, informal pharmacies, and urban slums, where most antibiotics are prescribed or sold as first-line, cost-effective care, primarily to avoid an out-of-pocket physician consultation fee.^3^ Furthermore, prophylactic health-seeking remains culturally marginal in SEA context, favouring reactive treatment post-illness. This gap between data-rich hubs and care-impoverished communities yields a syndemic condition. SEA region's prominence in contributing to global health metrics belies its limited capacity for actionable response.

Surveillance is not merely a technical process but a social act: it determines who and what becomes visible within the global governance.^1^^,^^4^^,^^5^ Limited technical capacity, constrained financial resources, weak regulatory systems, and slow behavioral change across all levels of health system collectively accelerate AMR and hinder the effective implementation of Antimicrobial Stewardship (AMS) programs.^2^ When laboratory data are not transformed into patient treatment or local empowerment of AMR control, it risks reinforcing a form of data extractivism, where countries are encouraged to generate data for global accountability without equivalent investment in diagnostic infrastructure or therapeutic access.^1^^,^^6^ It reflects a dual reality, though the expansion of surveillance system signal progress, it also simultaneously exposes inequities in health system across the region. Efforts should prioritize strengthening the underlying socio-economic and health-system infrastructure and quality of care.^7^

Access to “qualified medical care” in the region is also constrained with 6–7 doctors per 10,000 population, coupled with the difficulties in access to diagnostic facilities, prescribing or buying antibiotics is considered much easier and convenient.^4^^,^^8^ This hegemony, in which medicines substitute for medical care, has been described by social scientists as the *“pharmaceuticalisation”* of public health.^4^^,^^9^ This process places medicine at the core of modern life, transforming human conditions, capabilities, and capacities into opportunities for pharmaceutical intervention. In doing so, it reconfigures wider social dynamics, power relations, and cultural understandings of health, illness, and the body.^9^

Antibiotics function as “moral economy of care*”* an implicit social contract between the existing healthcare system and its patients which prioritizes immediate action over clinical rationality. Thus, what global health frameworks label as “irrational use” often symbolizes rational responses to systemic precarity.^5^ From a medical anthropological perspective, dependence on antibiotics is substitute for clean water, sanitation, and hygiene, diagnostic tools, and equitable healthcare.^2^^,^^5^^,^^7^^,^^10^

In SEA region, antibiotics are twisted into the health norms of people, sustaining both biological life and social expectations.^11^^,^^12^ Studies show that health workers in SEA region consistently highlight how diagnostic constraints, patient expectations, stock management pressures, weak regulatory environments with respect to infection prevention and control, and gaps in national treatment guidelines collectively drive the inappropriate prescription of antibiotics.^12^^,^^13^ This demonstrates a structural barrier that extends far beyond individual prescribing practice.^12^^,^^13^ Sustainable control of AMR requires interventions that address more than the prescribing behaviour of physicians.^10^ If antibiotics are to be preserved as a global public good, the response must extend beyond stewardship and surveillance to achieving community's trust, care, and access.

AMR must be understood and tackled as a syndemic phenomenon - arising from interacting, co-present, or sequential diseases which are shaped by the social, cultural, political economy and environmental conditions that promote and intensify resistance (Fig. 1).^14^^,^^15^

**Fig. 1:**
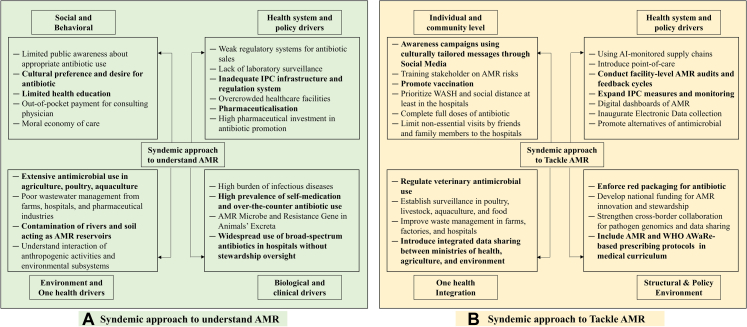
Proposed syndemic approach to understand (A) and tackle (B) AMR in Southeast Asia. AMR, Antimicrobial Resistance; IPC, infection prevention and control; WASH, Water, Sanitation, and Hygiene; WHO, World Health Organization; AWaRe, Access, watch and Reserve group of antibiotics according to the World Health Organization guideline.

The success of AMR governance should be measured not only by data completeness but by equity of outcomes guided by the attainment of universal health coverage. Bridging this gap requires not another layer of metrics, but a reorientation of moral and material priorities from surveillance to solidarity, from global measurement to mutual responsibility.

To confront AMR in the region, it is necessary to reform the social phenomenon of over-reliance on antibiotics and restructure the health care system. The growing scientific capacity, community engagement, and policy momentum places the region in a position to redefine what effective and equitable AMR governance can look like. To combat AMR, the world needs to invest not only in data, but in dignity; not only in metrics, but in prophylactic medical care that could prevent infection.

## Contributors

SB conceptualised the Commentary and developed the first draft. SB and FC critically reviewed, revised, and approved the final draft.

## Declaration of generative AI and AI-assisted technologies in the writing process

During the preparation of this work, we used Grammarly AI to assist with grammar checking and language refinement. After using this tool, the authors reviewed and edited the content as needed and take full responsibility for the final content of the publication.

## Declaration of interests

The authors declare no conflicts of interest.
